# [Retracted] miR‑206 inhibits cancer initiating cells by targeting EHF in gastric cancer

**DOI:** 10.3892/or.2023.8634

**Published:** 2023-09-25

**Authors:** Yan-Cui Gong, Guo-Liang Ren, Bo Liu, Feng Li, Hong-Peng Zhao, Jing-Bo Chen, Yu-Peng Li, Hai-Hua Yu

Oncol Rep 38: 1688–1694, 2017; DOI: 10.3892/or.2017.5794

Following the publication of this paper, it was drawn to the Editor's attention by a concerned reader that the colony formation assay data shown in Figs. 2, 4 and 8 were strikingly similar to data that had already appeared in another article written by different authors at different research institutes [Chen W, Wang J, Liu S, Wang S, Cheng Y, Zhou W, Duan C and Zhang C: MicroRNA-361-3p suppresses tumor cell proliferation and metastasis by directly targeting SH2B1 in NSCLC. J Exp Clin Cancer Res 35: 76, 732516, 2016].

Owing to the fact that the contentious data in the above article had already been published prior to its submission to *Oncology Reports*, the Editor has decided that this paper should be retracted from the Journal. The authors were asked for an explanation to account for these concerns, but the Editorial Office did not receive a reply. The Editor apologizes to the readership for any inconvenience caused.

